# Comparison of Mothur and QIIME for the Analysis of Rumen Microbiota Composition Based on 16S rRNA Amplicon Sequences

**DOI:** 10.3389/fmicb.2018.03010

**Published:** 2018-12-13

**Authors:** Adrian López-García, Carolina Pineda-Quiroga, Raquel Atxaerandio, Adrian Pérez, Itziar Hernández, Aser García-Rodríguez, Oscar González-Recio

**Affiliations:** ^1^Departamento de Mejora Genética Animal, Instituto Nacional de Investigación y Tecnología Agraria y Alimentaria, Madrid, Spain; ^2^Departamento de Producción Animal, NEIKER-Tecnalia, Vitoria-Gasteiz, Spain; ^3^Departamento de Producción Agraria, Escuela Técnica Superior de Ingeniería Agronómica, Alimentaria y de Biosistemas, Universidad Politécnica de Madrid, Madrid, Spain

**Keywords:** mothur, QIIME, 16S rRNA, rumen microbiota, bovine

## Abstract

**Background:** Microbiome studies need to analyze massive sequencing data, which requires the use of sophisticated bioinformatics pipelines. Up to date, several tools are available, although the literature is scarce on studies that compare the performance of different bioinformatics pipelines on rumen microbiota when 16S rRNA amplicons are analyzed. The impact of the pipeline on the outcome of the results is also unknown, mainly in terms of the output from studies using these tools as an intermediate phenotype (pseudophenotypes). This study compares two commonly used software (Quantitative Insights Into Microbial Ecology) (QIIME) and mothur, and two microbial gene data bases (GreenGenes and SILVA) for 16S rRNA gene analysis, using metagenome read data collected from rumen content of a cohort of dairy cows.

**Results:** We compared the relative abundance (RA) of the identified OTUs at the genus level. Both tools presented a high degree of agreement at identifying the most abundant genera: *Bifidobacterium, Butyrivibrio, Methanobrevibacter, Prevotella*, and *Succiniclasticum* (RA > 1%), regardless the database. There were no statistical differences between mothur and QIIME (*P* > 0.05) at estimating the overall RA of the most abundant (RA > 10%) genera, either using SILVA or GreenGenes. However, differences were found at RA < 10% (*P* < 0.05) when using GreenGenes as database, with mothur assigning OTUs to a larger number of genera and in larger RA for these less frequent microorganisms. With this database mothur resulted in larger richness (*P* < 0.05), more favorable rarefaction curves and a larger analytic sensitivity. These differences caused significant and relevant differences between tools at identifying the dissimilarity of microbiotas between pairs of animals. However, these differences were attenuated, but not erased, when SILVA was used as the reference database.

**Conclusion:** The findings showed that the SILVA database seemed a preferred reference dataset for classifying OTUs from rumen microbiota. If this database was used, both QIIME and mothur produced comparable richness and diversity, and also in the RA of most common rumen microbes. However, important differences were found for less common microorganisms which impacted on the beta diversity calculated between pipelines. This may have relevant implications at studying global rumen microbiota.

## Introduction

Research on ruminal microbiota is becoming increasingly important in dairy cattle as the microbial communities and their genome expression are related to important traits as health condition (Zilber-Rosenberg and Rosenberg, 2008), feed enteric fermentation (Zhou et al., 2009, 2010), or methane emissions (Wallace et al., 2015; Kamke et al., 2016; Roehe et al., 2016). The differences in the microbiota composition have also been proposed as a predictor or proxy of the differences in complex traits and environmental phenotypes (Ross et al., 2013; Kamke et al., 2016). Improving these traits is relevant for farm profitability and sustainability (Basarab et al., 2013; Bell et al., 2013; González-Recio et al., 2014a). Further, there is increasing interest on inferring the host genetic influence on the microbiota composition (Goodrich et al., 2016; Roehe et al., 2016). Tools that accurately estimate the microbial composition are essential to associate microbiota to phenotype variability.

Advances in sequencing technologies allow for obtaining genomic information in a fast and affordable manner. Whole metagenome and rRNA amplicons sequencing provide useful information to characterize the microbial composition in a given environment. Metagenomic information from hypervariable regions in the 16S and 18S ribosomal RNA amplicons are so far preferred in microbiome research due to their lower cost and reasonable accuracy. The results of these kind of studies rely on computational tools that provide accurate characteristics from large data sets of DNA sequences from the community under investigation (Lindgreen et al., 2016). Several authors have reviewed the specifications of different bioinformatics tools to analyze 16S rRNA gene sequences (Lozupone et al., 2005; Nilakanta et al., 2014; Oulas et al., 2015). Among these tools, mothur (Schloss et al., 2009; Kozich et al., 2013) and Quantitative Insights Into Microbial Ecology (QIIME) (Caporaso et al., 2010) are currently two of the most used suits of tools to analyze sequencing information from rRNA amplicons. However, comparisons between these tools on real data sets are scarce. For instance, other authors performed a benchmark study in order to investigate the performance of several tools in terms of microbial taxonomy and function (Lindgreen et al., 2016). These authors applied the methods on synthetic whole sequence metagenomes, which aim to represent the complexities encountered in a non-specific environment. In that study, QIIME resulting on a high specificity at determining the genus level but low sensitivity, whereas mothur was not tested. A recent study evaluated QIIME and mothur in fecal samples collected from preterm infants, showing slight differences in terms of the effective number of genera, richness and relative abundance (RA) detected (Plummer and Twin, 2015). Up to the best of our knowledge, the performance of these tools has not been yet evaluated in aligning rumen metagenome samples to public amplicons databases. Rumen microbiota poses the difficulty that most species have not been yet isolated, and therefore gene data bases may lack of many of the species in the rumen.

The aim of this study was to compare the rumen microbiota composition resulted from two different software: mothur and QIIME, when aligned against GreenGenes (GG) or SILVA databases. The null hypothesis is that the software and data base used to determine the ruminal microbial composition do not impact the results and conclusion from rumen microbiota studies.

## Results

**FIGURE 1:**
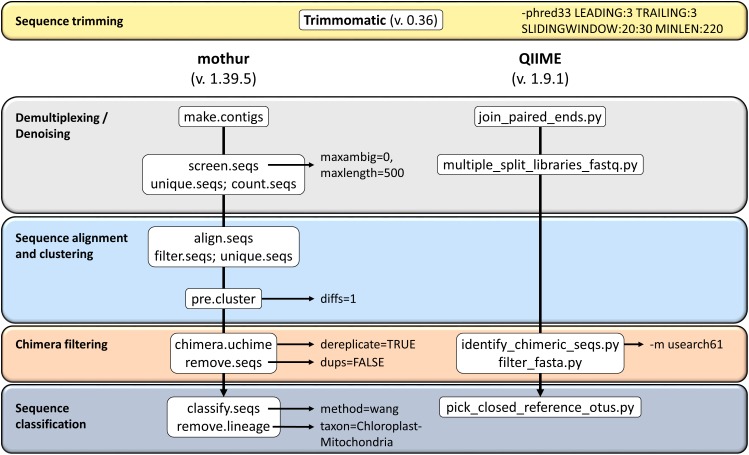
Overview of the workflows used in this study on QIIME and mothur for 16S rRNA amplicons analysis.

This study used sequence data from the hypervariable region V4 of the 16S amplicon from ruminal content in 18 dairy cows. The libraries were generated by means of Nextera kit. The 250 bp paired-end sequencing reactions were performed on a MiSeq platform (Illumina, San Diego, CA, United States). The sequences were processed using the two softwares: QIIME package version 1.9.1 (Caporaso et al., 2010) and mothur version 1.39.5 (Kozich et al., 2013). The RA of 16S rRNA gene reads for each sample and bioinformatics tool was used to infer the taxonomical composition of the samples, taking into account the copy number of 16S genes calculated from each tool. Two reference panels were considered for this purpose: GreenGenes (GG) database (May 2013 version) and SILVA (release 132). The detailed pipeline from each software is shown in Figure 1.

**FIGURE 2:**
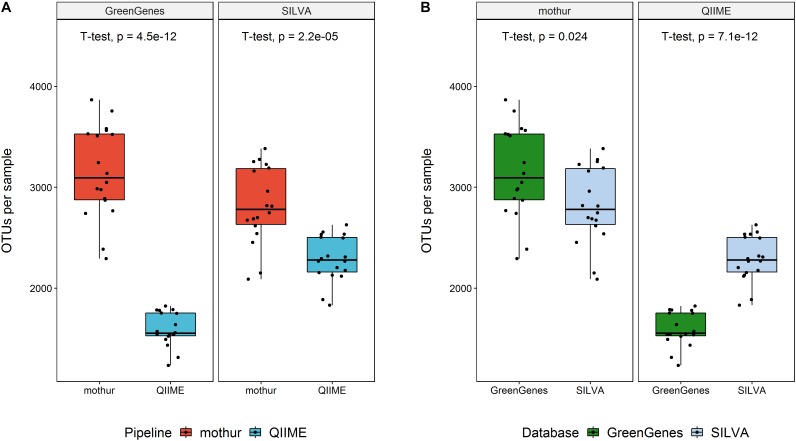
Number of OTUs per sample left for taxonomic classification within reference database (above) or within software (below).

After filtering and chimera removal, both tools used a similar number of sequences to cluster (*P* > 0.05), regardless the database used. In average, QIIME left 54,544 reads (*SD* = 9,041) per animal, whereas mothur worked with 53,790 reads per sample (*SD* = 7,709). However, mothur clustered these sequences in a larger number of OTUs regardless the database (Figure 2). Using QIIME with GG as reference database kept the lowest number of OTUs for classification.

### Taxonomical Richness

The performance of each tool was evaluated by looking at the assignment of individual OTUs and the number of genera classified. The RA of genera in each sample was calculated after excluding those genera that appeared at RA < 0.1% across all samples.

**FIGURE 3:**
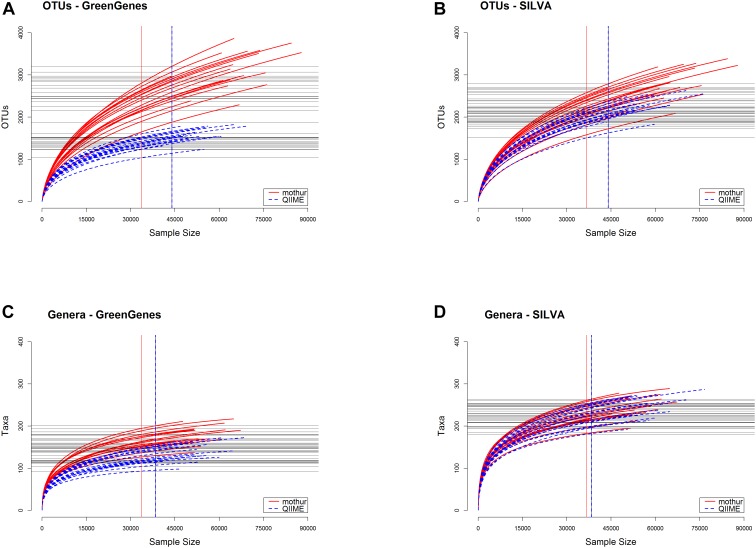
Rarefaction curves on OTUs **(A,B)** or classified genera **(C,D)** for the samples analyzed with each tool prior to filtering by relative abundance <0.1%. **(A,C)** represent curves from GreenGenes as the reference database, whereas **(B,D)** are obtained from SILVA database.

Figure 3 shows the rarefaction curves from each tool. Mothur detected a larger number of OTUs (Figures 3A,B) and also of microbial taxa at the genus level (Figures 3C,D) (*P* < 0.01) in the samples than QIIME using both GG and SILVA databases. Opposite, QIIME classified a larger number of genera after filtering by RA > 0.1% (Table 1). Most of the additional genera encountered by mothur were in very low abundance.

**Table 1:** Total of genera (N) and its average relative abundance (standard deviation within brackets) assigned by each tool (only by QIIME, only by mothur or by both).

Reference database	Total genera assigned			Relative abundance		
	QIIME	Mothur	Mothur and QIIME	QIIME	Mothur	Mothur and QIIME
GreenGenes	1	6	23	0.19 (-)^1^	2.89 (9.67)	2.60 (8.30)
SILVA	13	3	52	0.28 (0.13)	1.90 (6.51)	1.79 (5.67)

*Results from each reference data set are presented separately. ^1^No standard deviation calculated with n = 1.*

### Classification

#### GreenGenes

On average, mothur clustered a significantly (*P* < 0.001) higher number of OTUs per sample than QIIME. In average per sample, QIIME could not assign 61% (*SD* = 2.7) of clustered OTUs to a known genus, considering known every genus not named as “unclassified,” “uncultured,” “ambiguous,” “unidentified,” “unknown,” or null, whereas mothur could not assign a larger proportion (67%, *SD* = 2.5) of OTUs. QIIME was less restrictive at assigning OTU to genus level (*P* < 0.001), which might be related with the higher initial number of OTUs clustered by mothur, as we mentioned before. With this database, mothur identified a total of 29 different genera appearing in more than one sample, whereas QIIME assigned 24. Twenty three of these genera were common to both pipelines. The former aligned sequence data to six additional known genera, although most of them appeared in an average RA lower than 0.5%. Three out of these six genera had low representativeness, appearing in less than four out of 18 samples. On the other hand, the only QIIME-exclusive genus, *Bacillus*, had a low average RA and low representativeness, appearing only in three samples. Table 1 shows the average RA of genera assigned by one or both tools, highlighting that reads that were assigned to a known genus by only one of the tools appeared in very low RA. Both tools were capable of assigning around 99% of reads to any known taxonomy rank belonging to either bacteria or archaea kingdoms.

**FIGURE 4:**
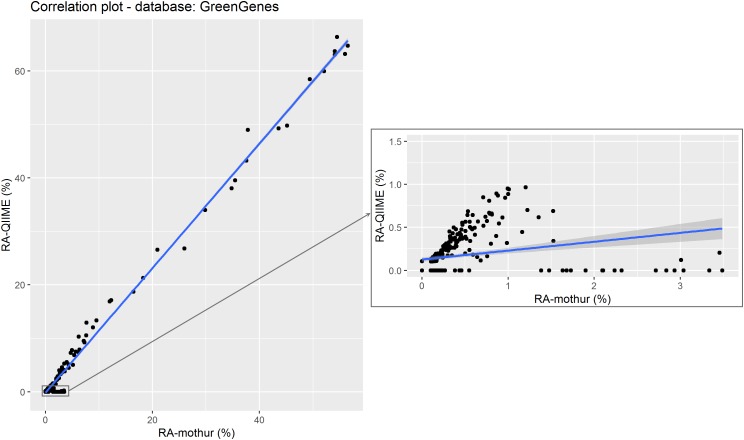
Relative abundance of the different microorganisms (by genera) detected by QIIME and mothur within the 18 samples using GreenGenes as reference data set. The subset shows the correlation between data with QIIME RA < 1%. Points represent individual RA within each sample.

A scatter plot of the RA estimated by each tool for each genus within sample are shown in Figure 4. A strong Pearson correlation (0.996; *P* < 0.001) was found between RA obtained from each tool. Although some small variability can be seen for some samples, there were not statistical differences in the overall RA between tools (*P* > 0.05). However, this disagreement was more evident for microorganisms at RA < 10%, for which significant differences were found between both tools (*P <* 0.05), and these differences were even higher at RA < 1% (*P* < 0.01), and the regression coefficient of RA from QIIME on RA from mothur differed from one, becoming even lower when subsetting the RA dataset (Table 2).

**Table 2:** Regression (slope and regression coefficient estimates) of the relative abundance from QIIME on the relative abundance from mothur using GreenGenes or SILVA as reference data set.

	GreenGenes		SILVA	
Reference population	Coefficient	*R*^2^	Coefficient	*R*^2^
All	1.10**	0.90**	1.14**	0.98**
Relative abundance < 10%	0.50**	0.31**	0.86**	0.84**
Relative abundance < 1%	-0.03**	0.05**	0.12**	0.14**

*^∗∗^P < 0.001.*

#### SILVA

Mothur also clustered a significantly higher number of OTUs in known taxa than QIIME (*P* < 0.001) when SILVA was the reference database even though mothur filtered out a larger number of reads, but the differences were more attenuated than using GG. These OTUs from mothur were nonetheless classified into a lower number of known taxa than using QIIME (Table 1). Both tools identified a total of 52 known genera. Mothur aligned sequence data to three additional exclusive known genera that appeared in more than 1 sample, and QIIME identified 13 genera that did not appeared in mothur. With SILVA as database, mothur could not assign an average of 36.1% per sample (*SD* = 1.37) of clustered OTUs to a known genus, but with QIIME only 9.1% (*SD* = 1.36) of OTUs were not assigned to known genera. Thus, mothur appeared to be much more restrictive (*P* < 0.001) at assigning OTUs to genus level when SILVA was used as the reference database.

**FIGURE 5:**
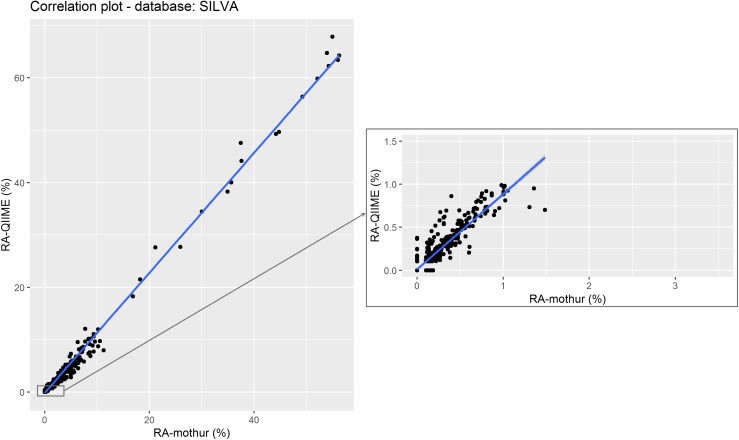
Relative abundance of the different microorganisms (by genera) detected by QIIME and mothur within the 18 samples using SILVA as reference data set. The subset shows the correlation between data with QIIME RA < 1%. Points represent individual RA within each sample.

Figure 5 shows a scatter plot of the RA estimated by each tool. As in the previous case for GG, a strong correlation (0.996; *P* < 0.001) was found between RA obtained from each tool. However, the regression coefficient of RA from QIIME on RA from mothur deviated from 1 at RAs < 10%, although smaller differences were observed compared to GG (Table 2). This suggests that mothur detects larger RA of microbes that are present in lower proportion in the rumen.

In summary, both tools were able to classify microorganism from the following genus: *Prevotella, Butyrivibrio, Succiniclasticum, Methanobrevibacter, Treponema, Bifidobacterium, Pseudobutyrivibrio, Ruminococcus, Mogibacterium, Lachnospira, Acetobacter, Methanosphaera* and *Desulfovibrio*, regardless the database. In addition, other microbes were not identified to genus level, but as members of *Lachnospiraceae* and *Ruminococcaceae* families, regardless the database. The OTUs unable to be classified by QIIME at the genus level were from the *Paraprevotellaceae, Coriobacteriaceae, Prevotellaceae*, and *Succinivibrionaceae* families when GG was used as the reference database and from the *Christensenellaceae* family when SILVA was used as the reference dataset. The OTUs that were not assigned to a genus level by mothur belonged to *Enterobacteriaceae* and *Spirochaetaceae* families using GG, and to *Bacillaceae, Enterobacteriaceae, Erysipelotrichaceae, Family_XIII, Prevotellaceae*, and *Spirochaetaceae* families using SILVA. Also, members from *Bacteroidetes, Firmicutes*, and *Proteobacteria* phyla were not assigned to family level when using mothur, regardless the database.

The genera that were identified exclusively by either mothur or QIIME are shown in Table 3. This table includes the reference database they were detected with, and previous studies reporting these microbes in rumen microbiota. Other genera were classified by both tools, but not for both databases. Among those with RA > 0.5% we found *YRC22* and *Clostridium* when GG was the reference database, and *Acetitomaculum, Saccharofermentans, Schwartzia, Candidatus_Saccharimonas* and some groups from families *Ruminococcaceae, Christensenellaceae, Rickenellaceae, Lachnospiraceae*, and *Prevotellaceae* when SILVA was used. Five taxa were identified for any combination of tool and database that have not been reported in rumen so far: *Eubacterium hallii group, Eubacterium_nodatum_group, Ruminococcaceae UCG-011, Ruminococcus gauvreanii group*, and *Prevotella P9*.

**Table 3:** Genera identified exclusively by mothur or QIIME, their function or activity in the rumen (if known), the reference database it was identified from, and information source or reference.

Genus	Function/activity in rumen	Reference database	Tool	Previous source(s)
*p-75-a5*	Detected in ruminal liquid fraction	GreenGenes	mothur	Jewell et al., 2015
*SHD-231*	Detected in rumen. Reduced in diets containing linseed diets	GreenGenes	mothur	de Carvalho et al., 2017
*Lachnospira bacterium FD2005*	Detected in rumen	SILVA	mothur	Azevedo et al., 2015.
*Papillibacter*	Detected in rumen. Cellulose- degrading bacteria	SILVA	mothur	Zhang et al., 2014
*Ruminococcus*	Cellulolytic bacteria	SILVA	QIIME	Wallace et al., 2015.
*Bacillus*	Amylolytic bacterium	GreenGenes, SILVA	QIIME	Gallo et al., 2016
*Eubacterium_ cellulosolvens_group*	Fibrolytic (Sika deer) Detected in sheep rumen	SILVA	QIIME	[Bibr B21][Bibr B2]
*Eubacterium_coprosta noligenes_group*	Detected in rumen	SILVA	QIIME	[Bibr B35][Bibr B27]
*Eubacterium_ ruminantium_group*	Present in rumen with an appropriate balance of degradable protein and carbohydrates	SILVA	QIIME	Abdelmegeid et al., 2018
*Eubacterium_ ventriosum_group*	Present in forestomach (Alpacas and Sheep)	SILVA	QIIME	Abdelmegeid et al., 2018
*Lachnospiraceae_ NK4A136_group*	Detected in rumen	SILVA	QIIME	Azevedo et al., 2015
*Roseburia*	Adherent bacteria community involved in plant degradation	SILVA	QIIME	Huws et al., 2016

### Diversity

**FIGURE 6:**
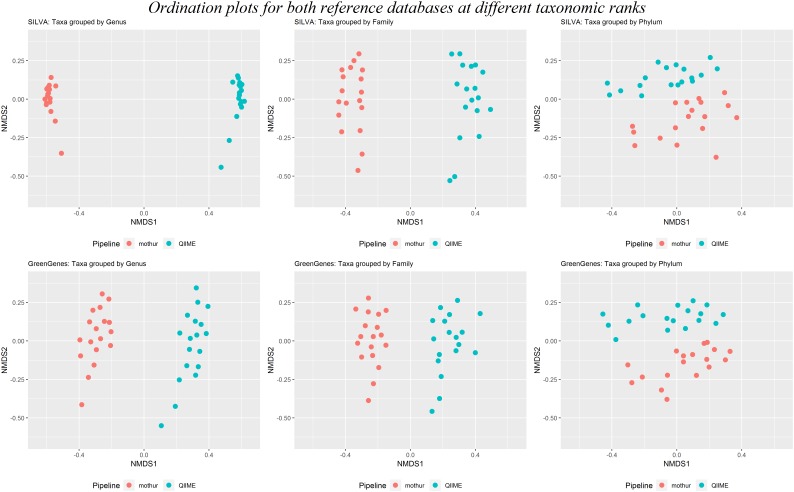
Two first dimensions of a non-metric multidimensional scaling for samples analyzed with mothur (red dots) or QIIME (blue dots) using either SILVA (above) or GreenGenes (below) as the reference dataset at the genus, family, and phylum taxonomical levels.

Beta-diversity was calculated to investigate the dissimilarity between rumen microbiotas within tool. Results clearly clustered by software at taxonomical levels of genus, family and Phylum, regardless the reference database used (Figure 6). This figure also shows that the dissimilarities between samples were larger between than within software at lower taxonomic levels (genus and family), whereas distances at the phylum level were similar between and within software.

Computation requirements were not reported here as they greatly depend on the computational strategy applied in terms of parallelization, available number of cores, and the bioinformatician’s creativity to design more efficient pipelines.

## Discussion

The results of this study support previous research showing differences between bioinformatics tools analyzing 16S rRNA amplicons. The number of OTUs and the taxonomic classification resulted thereof was compared between mothur and QIIME.

The results herein show that the final number of taxa and their overall RAs are not statistically different between tools using SILVA as reference database, but beta-diversity between samples clustered together by software used. Mothur worked with a larger number of OTUs, and these were classified into a larger number of genera than by QIIME when GG was the reference database. Plummer and Twin (2015) showed larger richness (total number of different genera) using QIIME compared to mothur, using the same reference database for alignment, although they used human preterm gut samples. They also showed statistical differences between tools on the comparison for diversity within samples. Our results show that using more updated SILVA releases mitigated these differences in terms of richness and diversity, suggesting that not only the implemented pipeline/program strongly influences diversity results, but database should also be considered in microbiota analysis. Therefore, it must be pointed out that comparison between pipelines in terms of number of OTUs must be done within database, and in terms of number of OTUs remaining for classification. It is also worth to highlight that in the current study both pipelines utilize a reference database for chimera filtering as well as a differentiated OTU labeling, making the comparison for raw OTUs unfeasible. Analyzing OTUs instead of taxonomical levels might be of interest in some circumstances. The results at the taxa level showed differences between tools and databases, which may be extended at the more specific level of OTUs.

It must be pointed out that the objective of this study was not to determine what tool provides a more accurate picture of the true microbiota composition, since the latter is unknown in cultivated rumen samples. There is no gold standard microbiota with known composition as many of the microbes in the rumen cannot be isolated or have not been yet cultivated. Sequencing the 16S rRNA gene poses the limitation that closely related microbes can be indistinguishable as they harbor almost identical sequences at this amplicon, and the different tools handle these drawbacks differently. A favorable pipeline should maximize sensitivity with a minimum impair in specificity. According our results, we infer that mothur provides higher diversity than QIIME regardless the reference database. There were five taxonomy groups detected in our study that have not been reported in rumen microbiota before. The rumen microbiota is largely underrepresented in databases and most of them have not been cultured yet (Stewart et al., 2018). Therefore, we consider that these groups of microorganisms might be considered as new candidates, although it must be corroborated in future studies through deep sequencing analysis or culture isolation. If they are true positive, they may be potential candidates to create mock communities to challenge bioinformatics tools. Lindgreen et al. (2016) showed low sensitivity scores and an impaired prediction performance with QIIME using a benchmark metagenome. However, as noted by these authors, QIIME uses custom databases that only contain specific marker sequences such as 16S rRNA. Therefore, performance of QIIME in whole metagenome analyses cannot be extrapolated to 16S or 18S amplicons studies. In addition, mothur was not used in such a study.

There are two main differences between mothur and QIIME: the OTU clustering algorithm and the algorithm for taxonomic classification. The alignment and clustering processes differ between software, as well as the chimera detection. Mothur handles the taxonomic classification using a naïve Bayesian classifier using a pseudo-bootstrapping to generate a confidence score, which must be over 80% to assign a read to a given taxonomy (Wang et al., 2007). QIIME uses the usearch algorithm to find the closest match in a reference data base (Edgar, 2010), which has been reported problematic at identifying the closest reference because it is sensitive to the order of the reference sequences as they can be identical over the region being considered (Westcott and Schloss, 2015). Usearch shows a high level of sensitivity to detect reference sequences, however, the specificity of those matches was poor relative to the true best match. High error rates have been previously reported with GG, and could be substantially improved by randomizing the sequences (Westcott and Schloss, 2015). Further the poorly GreenGenes-aligned sequences artificially increases the distances between sequences, which may also impair the accuracy of the classification. QIIME uses a closed reference pick up strategy in a single step, which implies some difficulties at disentangling all dissimilarities with mothur. The way mothur is implemented here resembles a pseudo open-reference pick up, because there were a previous step of chimera filtering and a known reference database was used for classification. These arguments might explain the poorest performance of QIIME in our data set when GG was used as the reference data set.

This is a proof of principle analysis showing how the choice of bioinformatics pipeline and the reference data set can impact the analysis of 16S rRNA gene sequencing data from rumen microbiotas. Nonetheless, the bio-informatics tools could perform differently in samples from different sites as different body parts may host different taxonomic composition, making the algorithms more or less efficient at detecting the true composition.

In the light of the results obtained in this study we can conclude that the impact of the tool is relatively small in terms of richness as a more updated and comprehensive reference database is used. SILVA seems to be a preferred reference data set as a larger number of different genera were identified, and more consistent results were obtained between tools. SILVA is a more updated database, whereas GG has not been updated since 2013. However, differences were detected in terms of beta-diversity, and differences between pipelines were obtained for microbes in lower abundances, yet belonging to the core microbiome. In this sense, mothur showed larger sensitivity at detecting microorganisms that can potentially populate the cow rumen. This may be important, as differences in the RA of less frequent groups of microbes may be relevant. These differences affect the microbiota similarity between samples or individuals. In turn, this would affect the phenotypic variance of a complex trait explained by the microbiota using mixed models that accounted for the microbiota composition as a random effect with a covariance structure given by these similarities between samples. Performance of mixed model methodology under Best Linear Unbiased Prediction or any other Reproducing Kernel Hilbert Space scenario greatly depends on the structure of the covariance or kernel matrix used as reported in González-Recio et al. (2009, 2014b). Incorrect or improper microbiotas similarity matrices might bias the proportion of variance explained by the microbiota or genetic correlation estimates between host genome and its metagenome. Up to the best of our knowledge, there is no proof of concept to determine what tool provides a more suitable similarity matrix. The degree of aimed microbiota specificity may affect the choice of the pipeline. Mock communities that mimic the true composition of rumen microbiota are not yet available. This study also highlights the necessity to create benchmark samples with a known composition of cultivated ruminal microorganisms to evaluate different bioinformatics tools, as well as the convenience of including more rumen specific communities into the gene databases. In this sense, those mock samples could include the genera that have been detected by only one of the tools (Table 3). Moreover, It must be consider that the samples used in this study combined the four possible ruminal fractions and the RAs in the samples might differ from the true composition in the rumen. Nonetheless, this is not expected to affect the comparison between pipelines.

## Materials and Methods

This study was carried out in accordance with Spanish Royal Decree 53/2013 for the protection of animals used for experimental and other scientific purposes. An ethics committee was not necessary in this case because it was conducted on pre-existing data from a previous trial based on routine management practices in commercial farms. Data used in this study were described in Gonzalez-Recio et al. (2018). In brief, samples were obtained from ruminal content from 18 cows from 2 breeds (10 Holstein and 8 Brown Swiss) allocated in the Fraisoro Farm School (Zizurkil, Gipuzkoa, Spain). Ruminal samples were collected from each dairy cow using a stomach tube connected to a mechanical pumping unit. About 100 ml of each ruminal extraction were placed into a container and were frozen immediately after the extraction and then stored at -20 ± 5°C until analysis. Samples were gradually thawed overnight at refrigeration (5 ± 3°C) and squeezed through four layers of sterile cheesecloth to separate solid (solids with a particle size smaller than the diameter of the sampling tube) from liquid digesta phases. This latter phase was subsequently separated into planktonic organisms and bacteria associated with the liquid fraction. The solid phase was separated in associated and adherent fractions. Fractionation procedures were carried out following the methodology described in Yu and Foster (2005). The four fractions were lyophilized and combined to obtain a unique sample with the four fractions represented proportionally (on dry matter basis).

After composition, DNA extraction was performed using the commercial Power Soil DNA Isolation kit (Mo Bio Laboratories, Inc., Carlsbad, CA, United States) following manufacturer’s instructions. The extracted DNA was subjected to paired-end Illumina sequencing of the V4 hypervariable region of the 16S rRNA. Universal bacterial 16S rRNA gene primers (515F: 5′-GTGCCAGCMGCCGCGGTAA-3′ and 806R: 5′-GGACTACHVHHHTWTCTAAT-3′ (Caporaso et al., 2011) were used to generate the bacterial amplicon libraries (expected amplicon size 250 bp). The libraries were generated by means of Nextera kit. The 250 bp paired-end sequencing reactions were performed on a MiSeq platform (Illumina, San Diego, CA, United States). Data are publicly available at http://www.ebi.ac.uk/ena/data/view/PRJEB26635.

Sequences were pre-processed using Trimmomatic tool (v 0.36) (Bolger et al., 2014). Sequences below 220 bp in length and average quality score below 30 on a window of 20 bases were discarded. In total, 3,261,168 reads were analyzed. The remaining sequence data were then processed using the two softwares: QIIME package version 1.9.1 (Caporaso et al., 2010) and mothur version 1.39.5 (Schloss et al., 2009; Kozich et al., 2013). In the case of QIIME, forward and backward reads were joined with join_paired_ends.py. Chimeras were identified and filtered using *usearch* method (Rognes et al., 2016). Finally, the tool was used to pick closed-reference OTUs from the GreenGenes database (May 2013 version) or SILVA database (Quast et al., 2013)^1^ (release 132) and representative sequences with a 99% of similarity were kept. The pipeline for mothur also began by joining forward and backward reads. Chimeras and unique sequences were removed using UCHIME (Edgar et al., 2011). Sequences were then preclustered, and finally classified using the default method (naïve Bayesian classifier; Wang et al., 2007) on *classify.seqs()*, with the same cut-off for sequence identity and reference databases as above. OTUs were summarized at phylum, class, order, family, and genus. Phylogenetic groups with an abundance lower than 0.1% in all samples were excluded from the final analyses. The pipelines used can be found in a git-hub repository^2^.

All statistical analyses were performed in R v3.5.1 (R Core Team, 2015). When Pearson correlation was calculated, the statistical significance was tested using the cor.test() command from the base package.

### Filtering and Chimera Removal

Differences in the number of sequences left after chimera removal from each tool (mothur vs. QIIME) was computed using a least squared mean regression.

The linear model was:

y=μ+xβ+e

where **y** was the vector of the number of reads left for each sample after filtering and chimera removal with either mothur or QIIME (*n* = 2 × 18), μ is the intercept, **x** is the incidence vector assigning each record to the corresponding tool (mothur vs. QIIME), β is the coefficient estimate, and **e** is the vector of residuals assumed to be independently and identically normally distributed. The level of significance was set to α = 0.05.

### Richness and Relative Abundance

Differences between mothur vs. QIIME were computed using a simple generalized linear model. Sequence reads from each sample (*n* = 18) were analyzed with the mothur or QIIME pipelines, and using either SILVA or GG databases. The statistical analysis for the resulting richness and RAs were computed within database as follows:

y=μ+xβ+e

where **y** was the vector of number of microbial taxa at the genus level (or their RA) assigned either with mothur or QIIME (*n* = 2 × 18) using GG, μ is the intercept, **x** is the incidence vector assigning each record to the corresponding tool (mothur vs. QIIME), β is the coefficient estimate, and **e** is the vector of residuals assumed to be independently and identically normally distributed. The level of significance was set to α = 0.05.

Further, the same statistical analysis was performed using the RAs obtained with SILVA as the reference data base.

Similarly, the number of unclassified reads from each tool within reference database were analyzed using the same model as above.

### Dissimilarity Matrix and Principal Component Analyses

Non-metric multidimensional scaling (nMDS) was performed to explore the ruminal community structure, using the phyloseq package (v 1.24.2). The ordinate function was used to estimate dissimilarities using Bray–Curtis distances. Plot_ordination was used to plot these dissimilarities between mothur and QIIME pipelines with either SILVA or GreenGenes as the reference databases, grouping taxa by genus, family, and phylum levels.

## Author Contributions

AL-G, AP, and IH selected the steps in the pipelines for mothur and Qiime and analyzed the sequence files. AG-R, CP-Q, and RA executed the animal experiments, collected and analyzed the samples, discussed the results and helped to write the manuscript. AL-G and OG-R implemented the statistical analyses. OG-R designed the experiment and wrote the first draft of the manuscript. AL-G, CP-Q, RA, AG-R, and OG-R discuss the results. All authors read and approved the final manuscript.

## Conflict of Interest Statement

The authors declare that the research was conducted in the absence of any commercial or financial relationships that could be construed as a potential conflict of interest.

## Footnotes

1 http://www.arb-silva.de

2 https://github.com/alopgar/16S-analysis/tree/master/16S-mothur-and-QIIME-pipeline-comparison
